# Prevalence of cardiovascular risk factors in a middle-income country and estimated cost of a treatment strategy

**DOI:** 10.1186/1471-2458-6-9

**Published:** 2006-01-19

**Authors:** Pascal Bovet, Conrad Shamlaye, Anne Gabriel, Walter Riesen, Fred Paccaud

**Affiliations:** 1 Ministry of Health and Social Services, Victoria, Seychelles; 2 University Institute of Social and Preventive Medicine, Lausanne, Switzerland; 3 Institute of Clinical Chemistry and Hematology, Kantonspital, St Gallen, Switzerland

## Abstract

**Background:**

We assessed the prevalence of risk factors for cardiovascular disease (CVD) in a middle-income country in rapid epidemiological transition and estimated direct costs for treating all individuals at increased cardiovascular risk, i.e. following the so-called "high risk strategy".

**Methods:**

Survey of risk factors using an age- and sex-stratified random sample of the population of Seychelles aged 25–64 in 2004. Assessment of CVD risk and treatment modalities were in line with international guidelines. Costs are expressed as US$ per capita per year.

**Results:**

1255 persons took part in the survey (participation rate of 80.2%). Prevalence of main risk factors was: 39.6% for high blood pressure (≥140/90 mmHg or treatment) of which 59% were under treatment; 24.2% for high cholesterol (≥6.2 mmol/l); 20.8% for low HDL-cholesterol (<1.0 mmol/l); 9.3% for diabetes (fasting glucose ≥7.0 mmol/l); 17.5% for smoking; 25.1% for obesity (body mass index ≥30 kg/m^2^) and 22.1% for the metabolic syndrome. Overall, 43% had HBP, high cholesterol or diabetes and substantially increased CVD risk. The cost for medications needed to treat all high-risk individuals amounted to US $45.6, i.e. $11.2 for high blood pressure, $3.8 for diabetes, and $30.6 for dyslipidemia (using generic drugs except for hypercholesterolemia). Cost for minimal follow-up medical care and laboratory tests amounted to $22.6.

**Conclusion:**

High prevalence of major risk factors was found in a rapidly developing country and costs for treatment needed to reduce risk factors in all high-risk individuals exceeded resources generally available in low or middle income countries. Our findings emphasize the need for affordable cost-effective treatment strategies and the critical importance of population strategies aimed at reducing risk factors in the entire population.

## Background

Cardiovascular diseases (CVD) have become a leading cause of mortality and morbidity in developing countries and rates are expected to rise further over the next few decades [1-4]. In particular, it has been estimated that high blood pressure (HBP) accounts for as much as 5.0% of the total mortality in middle-income countries, tobacco for 4.0%, high cholesterol for 2.1% and obesity for 2.7% [5].

The increasing burden of CVD has important economic implications. CVD occurs typically at a younger age in developing than developed countries with important consequences such as loss of revenue at household level and loss of productivity at macroeconomic level. From a health system perspective, huge resources are needed for providing health care to large numbers of chronic patients for decades and for sustaining increasingly sophisticated equipment and more skilled and harder-to-replace workforce [6-8].

There are two approaches to reduce the burden of CVD [7]. The population strategy, that includes community-based programs and health promoting policies, recognizes that several modifiable CVD risk factors are widely distributed in the population and that small change in CVD risk among large numbers of people can reduce largely the incidence of CVD in the population. The alternative is to target "high risk" people, i.e. risk factors are screened in the population and persons with high risk of CVD are treated. These alternative strategies, "population" and "high risk", can of course be considered as complementary.

In this study, we examined the prevalence of risk factors in the adult population of Seychelles, a country that has experienced rapid socio economic development over the past few decades. We then estimated the direct cost of treatment of all high-risk individuals (i.e. medication and simple related medical follow-up). The interest of this case study is enhanced by the fact that Seychelles recently switched its medication procurement system toward a broader use of generic drugs and direct cost estimates can be estimated for both systems.

## Methods

The Republic of Seychelles is a group of islands in the Indian Ocean approximately 1800 km east to Kenya. A large majority of the population is of African descent. Health care (including medication) is available at no direct cost at the point of delivery to all residents through a National Health System while a few private doctors also provide services on a fee-paying basis. The gross domestic product (GDP) per capita increased from US$600 in 1976 to US$8492 in 2003. Annual government expenditure on health was US$307 per capita per year [10]. All deaths are registered in Seychelles and Vital Statistics indicate a life expectancy of 69 years in men and 76 years in women. AIDS and CVD account for respectively approximately 1% and 38% of total mortality [11].

A population-based survey of cardiovascular risk factors was conducted in 2004 under the auspices of the Ministry of Health of the Republic of Seychelles (Seychelles Heart Study III). The sampling frame consisted of a sex- and age-stratified random sample of the entire population aged 25–64. Eligible participants were selected from computerized data of a national population census in 2002 thereafter updated by civil status authorities. Eligible persons were invited to attend study centers on the 3 main islands through a personal letter and requested to be fasting since midnight when they attend. The survey was approved by the Ministry of Health after technical and ethical reviews. Participants were free to participate and gave written informed consent.

Smoking was defined as reported smoking at least one cigarette per day. Blood pressure was defined as the average of the last two of three measurements with a mercury sphygmomanometer taken at intervals longer than 2 minutes after the participants had been seating for at least 30 minutes. BP categories are defined along current guidelines [12-15]. Weight was measured with electronic medical scales (Seca, Germany) and height measured with fixed stadiometers (Seca). Body mass index (BMI) was calculated as weight divided by height squared (kg/m^2^) and obesity defined as BMI ≥30 kg/m^2^.

Blood was taken between 7:00 and 10:00 in the morning and on another day for the few participants who reported to be non-fasting. Serum was obtained within 2 hours of blood collection and immediately frozen to -20°C. Blood lipids were measured at the Canton Laboratory for Biochemistry and Haematology, St Gallen, Switzerland. Blood cholesterol was measured by an enzymatic colorimetric test using cholesterol esterase and cholesterol oxidase (CHOD-PAP, Roche) on a Hitachi 917 instrument(Roche). Triglycerides were measured with an enzymatic colorimetric test GPO-PAP, Roche) on a Hitachi 917 instrument (Roche). HDL-cholesterol was measured with a homogeneous enzymatic colorimetric test (HDL-C plus 2^nd ^generation, Roche) using Dextransulfate and PEG-modified enzyme on a Hitachi 917 instrument. Blood lipids categories are in line with current guidelines [16].

Fasting plasma glucose was measured with a point-of-care analyzer (Cholestec LDX, Hayward, USA) in all participants. If glucose was ≥5.6 mmol/l, an additional capillary measurement was performed within 10 minutes (Ascencia Elite glucometer, Bayer). The average of the two readings was considered. Diabetes was defined as fasting blood glucose ≥7.0 mmol/l [17]. The metabolic syndrome was defined along the American guidelines [16], which requires 3 or more of the following: increased waist (>102 cm in men and >88 in women), increased triglycerides (≥1.7 mmol/l), low HDL-cholesterol (<1.03 mmol/l in men and 1.29 in women), elevated BP (≥130/85 mmHg), and increased fasting blood glucose (≥6.1 mmol/l).

For HBP, medical treatment was assumed for persons at 'medium' or 'high' CVD risk, as defined in WHO guideline [12]. In assessing CVD risk, we considered the following associated risk factors: age ≥55 for men (≥65 for women), obesity, high blood cholesterol (≥6.2 mmol/l), and low HDL-cholesterol. Low HDL-cholesterol was defined as <1 mmol/l for both men and women [16].

When assessing CVD risk in diabetic patients, we considered HBP (≥140/90 mmHg or antihypertensive treatment) and the risk factors mentioned in the paragraph above [12]. We considered total cholesterol levels alone (i.e. without considering HDL-cholesterol) for the purpose of simplicity and assuming that the number of cases with elevated total cholesterol and high HDL-cholesterol (possibly not requiring treatment) would be similar to the number of cases with moderately elevated cholesterol and low HDL-cholesterol (possibly requiring treatment).

Consistent with efficacy and cost considerations [12-14,18], treatment for HBP was assumed to include a diuretic as first choice for mono-therapy in 80% of cases, a diuretic in all combinations, and an approach favoring low-dose combinations. The different combinations (bi, tri or tetra-therapy) assumed to be used for treatment of HBP across different CVD risk categories was estimated by consensus with local physicians. For diabetes, 20 units insulin were assumed to be used in 10% of cases of persons with glucose ≥10 mmol/l, in addition to oral anti-diabetic drugs, assuming that most persons with diabetes had type II diabetes. For the few cases with type I diabetes, who do not need oral treatment, the cost for oral diabetic drugs accounted for in our model was assumed to compensate for the larger cost of higher insulin dose needed in type I diabetes.

Costs of medications are provided for both 2004 and 2005 because, in 2005, the Ministry of Health adopted a new procurement system in favor of generic medications. Prices of blood tests assumed to be performed for medical follow-up were provided by the laboratory of the main hospital of Seychelles. We assumed the price of a medical visit in the public service as the average between the minimal cost charged by private doctors (SCR 75) and the amount of SCR10 corresponding to 15 minutes of a typical salary of general practitioners in the public service. This compromise recognizes an additional cost for services contributed by nurses, clerks and administrative staff in addition to medical visit time in the public service. The number of 4 consultations per year was assumed, consistent with standard practice for chronic patients who need renewal of prescriptions every three months in the public service.

Biochemical investigations assumed in our estimates included one series of basic tests per year for all cases with either HBP, diabetes or dyslipidemia (i.e. costs for screening in the population are not included). In addition, diabetic patients were assumed to have blood glucose measured quarterly and HbA1c measured biannually. The tests are available in public health centers in Seychelles.

The prevalence of risk factors has been tabulated by age and sex and overall estimates are weighted to the distribution of the new World Health Organization standard population [19]. Costs in the entire population are expressed in Seychelles rupees (SCR) per year. Costs per capita of the entire population per year (in US$; 1$ ~5 SCR) provide cost estimates that are independent of the size of the population. When estimating costs of the treatment strategy in the entire population, we assumed same risk factor prevalence and medication patterns in persons aged ≥65 years as in the population aged 25–64; a zero prevalence of risk factors in persons aged less than 25; and a same prevalence of risk factors in 2004 and 2005. Analyses were performed with Stata 8.2.

## Results

From the initial sex and age stratified sample of 1632 men and women aged 25–64, 69 were dead, had emigrated, or could not be traced. From the 1563 eligible persons, 1255 participated in the survey (80.2%). Participation was 67% among men aged 25–34, above 70% for men in other 10-year age categories and above 80% for women in all 10-year categories. Participation did not differ across study centers.

Table 1 shows the prevalence of main risk factors and diabetes by age and sex (adjusted to the WHO standard population). The prevalence of HBP increased sharply with age and was higher in men than women. The prevalence of BP ≥140/90 mmHg and BP ≥160/100 mmHg was 31.6% and 11.0%, respectively. Among persons with BP ≥140/90 mmHg, 49.1% were currently taking antihypertension treatment. Around half of men and women aged 25–64 had serum cholesterol that exceeded optimal levels (≥5.2 mmol/l). A quarter of both men and women had high cholesterol levels (≥6.2 mmol/l). Approximately one fifth of men or women had low serum HDL-cholesterol (<1 mmol/l). The prevalence of HDL-cholesterol <1.3 mmol/l was 48.9% in women. The prevalence of diabetes increased sharply with age with an overall prevalence of 9.3%. As many as 68.3% of women and 52.0% of men had BMI ≥25 kg/m^2 ^and 35% of women and 15% of men were obese (≥30 kg/m^2^). Smoking was reported by 30.8% of men and 3.9% of women. The prevalence of the metabolic syndrome (age 25–64) was high at 25.5% in women and 18.7% in men.

The prices of medications available in public health services in Seychelles were considerably lower in 2005 than in 2004 (Table 2), consistent with lower cost of generic drugs used in 2005 vs. 2004 (except for statins).

Table 3 shows the distribution of CVD risk categories across categories of HBP, increased levels of blood cholesterol and diabetes. The table also shows treatment assumed to be used for HBP, high cholesterol and diabetes. Noticeably, the prevalence of persons with HBP, diabetes or hypercholesterolemia (42.7%) was only slightly larger than the prevalence of HBP alone, indicating that these conditions tended to cluster in same individuals. The cost per capita per year for treating all affected persons at "medium" or "high" CVD risk, in 2004 and 2005 respectively, amounted to respectively $30.4 and $11.2 for hypertension; $21.5 and $3.8 for diabetes; and $32.7 and $30.6 for hypercholesterolemia. The total cost of medications needed to treat all three conditions in the entire population would be $45.6 per capita per year in 2004 and $84.6 in 2005. This emphasizes the large cost reduction that resulted from switching to generic drugs in 2005.

Table 4 shows the cost for selected services for medical care of HBP, diabetes and dyslipidemia. The cost of medical visits could amount to $8.2 while laboratory exams could cost an additional $14.4 (a total of $22.6). Since HBP is much more prevalent than diabetes or hypercholesterolemia and because the considered risk factors tend to cluster (71% of persons with diabetes and 50% of persons with hypercholesterolemia also had HBP), the cost of these selected services was largely driven by treatment of HBP.

## Discussion

This paper examines the prevalence of major risk factors in a developing country and the costs of one specific strategy to prevent CVD, i.e. the identification and drug treatment of persons at high risk. The analysis is restricted to direct costs of the intervention (medication and medical follow-up), an immediate and practical issue for health care providers and patients. The case of Seychelles is interesting because it is a middle-income developing country; the prevalence of risk factors is based on recent population data; at least one medication from all major medication classes are available in the public sector; and the national health system allows for providing treatment to all high-risk individuals.

The survey showed high levels of the major CVD risk factors in a rapidly developing middle-income country. Prevalence in 2004 can be compared with data of a similarly designed population survey in 1989 (20,21). Adjusted to the WHO standard population, the overall prevalence of HBP (BP ≥140/90) was lower in 2004 than 1989 (31.6% vs. 38.6%, population aged 25–64), probably reflecting the larger proportion of persons with HBP under treatment in 2004 than in 1989 (59.3% vs. 21.9%). The higher prevalence of overweight in 2004 than in 1989 can relate to rapid socio-economic development [22], with large increase in motorised transport and service-oriented economy. This may account for the higher prevalence of diabetes and, to some extent, hypercholesterolemia (both higher in 2004 than in 1989). The prevalence of smoking was substantially lower in 2004 than in 1989, consistent with sustained government tobacco control interventions, including high taxes on tobacco products, advertising ban, education programs, ratification of the Framework Convention of Tobacco Control in 2003 and comprehensive legislation enacted in 2005.

We used conservative assumptions in our estimation model and costs for a treatment strategy provided in our study are likely to correspond to minimal figures. Our assessment of CVD risk did not include several indicators of CVD that are difficult to quantify, e.g. risk factors such as sedentary habits or family history of CVD and other markers of risk (target-organ damage, associated clinical conditions except for diabetes, or established CVD). Our cut-off values for age (≥55 years for men), total cholesterol (≥6.2 mmol/l) or HDL cholesterol in women (<1 mmol/l) are less stringent than in other guidelines. Measurement of blood pressure on one single visit tends to overestimate the prevalence of hypertension [23] but this may be partially compensated by the use of blood pressure values unadjusted for antihypertensive treatment (i.e. another source of underestimation of an individual's CVD risk). Overall, it is likely that we assessed CVD risk conservatively so that medical treatment would be justified for most patients labeled in our study as 'medium' or 'high risk'. We also used conservative criteria for the nature and frequency of biochemical analyses assumed for medical follow-up of high risk patients in our estimation. In particular, we did not include urine analysis, electrocardiogram, fundoscopy and others tests usually found useful in the circumstances. Also, we did not include interventions, such as aspirin, intended mainly for secondary prevention of CVD.

The cost of medications accounted for approximately two thirds of the total cost of the treatment strategy. It follows that a decreased price of medications will reduce the total direct cost of the treatment strategy. We show that by using generic rather than proprietary medications, the cost of medications alone could be substantially reduced: from $30 to $11 per capita per year for hypertension and from $22 to $4 for diabetes. The cost of medication for dyslipidemia could potentially be similarly reduced if generic medications were used. Although new statins are more powerful than older ones, the majority of subjects with elevated blood cholesterol would get to target even with the older agents while only a minority of patients at very high risk would need the latter to reach recommended very low LDL targets.

The cost of basic medical follow-up for high-risk patients, both investigations and medical visits ($23 per capita per year) is less compressible and indeed likely to increase under the pressure of increasing patients' expectations and other factors such as rising health care personnel costs. This emphasizes the need for guidelines to ensure that utilization of scarce resources is not unduly influenced by local or global commercial interests but used in a cost-effective way [24].

At a minimum of $68 per capita, the estimated direct costs for medications and basic medical follow-up for all high-risk individuals is a major burden on the health budget. Even at half of that cost to account for half of the prevalence of risk factors found in this study -a situation more typical of populations at earlier stages of the epidemiological transition-, treating risk factors in the population would remain out of reach for many developing countries. Indeed, many developing countries rely on government health budget lower than $50 per capita per year -vs. $3000–5000 in western countries- [10] and a large proportion is absorbed to control the persisting large burden of infectious diseases. There has been much debate as to whether medical treatment for chronic conditions can be afforded in low resource settings, in view of high cost of life-long medication and, indeed, high cost per year of life saved compared to interventions for other conditions [25]. It has been argued that scarce resources could be better invested in more cost-effective or urgent medical or other interventions [26,27]. It would however be inequitable to deny treatment of demonstrated efficacy to some or all individuals who would benefit of it [28].

We considered costs in the public sector, which provides universal coverage for health care in Seychelles. We have not investigated or compared costs and prices in the private sector, which remains relatively small. Currently, the public services are financed from taxation revenue, an approach aimed at providing more equitable provision of care to the population. Increasing pressure for the introduction of user fees is likely to impact negatively on those who are most vulnerable and least able or willing to pay.

Furthermore, direct cost for medical treatment of elevated risk factors is of course only one component in the spectrum of medical care required and, more generally, in cost-benefit analysis. Costs can be averted as well, such as hospitalization and loss of productivity, therefore potentially reducing the overall cost of a high-risk strategy. In Seychelles, where some special care is available -e.g. hemodialysis but not revascularization-, the costs averted can be significant. However, these costs may not be immediate in many low and middle income countries, since hospitalization and surgery may not be available and low formal employment makes evaluation of the cost of lost productivity uncertain. Clearly, comprehensive cost-benefit analysis is a greater challenge in developing countries in the absence of documented evidence on the magnitude of such factors. While adopting a simplified approach, the analysis presented in this paper does provide approximate direct costs that must actually be borne in providing high-risk individuals with medication and care.

The high cost of a treatment strategy stresses the need to identify alternative and more affordable strategies. Emphasis has been put on the need to inform individual clinical treatment based on the probable size of absolute treatment benefits, i.e. based on an individual's CVD total risk, not just risk factor levels [29,30]. A major issue is to choose CVD risk thresholds that can maximize events prevented without increasing the total numbers treated in a defined population. However, assessing CVD risk is challenging in populations for which cohort data are not available and calculation of CVD risk along current western-based risk functions cannot be calibrated. A radically different strategy, recently proposed, would consist of providing a low-cost "polypill" combining several medications (e.g. three antihypertensive drugs, a statin, aspirin) to all persons aged 55 years and above, given that CVD risk is predominantly driven by age [31]. This approach may well be far less expensive than traditional treatment strategies because of expectedly much lower cost of such a polypill (use of generic drugs, economy of scale) and the avoidance of medical follow-up. However, clinical trials will have first to demonstrate the effectiveness, safety, and acceptability of such a strategy.

Even if drug prices are reduced, strategies relying on medications may still be unaffordable for many low or middle income countries. In addition, several factors inherently limit the impact of treatment strategies at a population level, e.g. poor compliance to medications for asymptomatic chronic conditions [32] and limited control of hypertension or diabetes in many instances, even with multi-drug combinations. Furthermore, approaches based on the assessment of lifetime CVD risk imply that individuals will have accumulated much of their risk (e.g. developed substantial atherosclerosis) before they become eligible for treatment [33].

Although a high-risk approach can be an important component of a global strategy to reduce CVD [34], evidence strongly supports the predominant role of primary prevention[35]. Interventions typically include policies aimed at changing the societal milieu to enable a broad adoption of healthy lifestyles [36]. In addition, by decreasing risk factor levels in the population (or maintaining favorable levels in some developing countries in early epidemiological transition), primary prevention can substantially minimize the number of persons at high CVD risk in need of medical treatment for hypertension, dyslipidemia or type II diabetes. Ultimately, the interests of the population at large are served by broad improvements in the quality of life, which includes both a reduction in morbidity as well as a decrease in fatal events. Since prevention delivers the best results in terms of quality of life, that should be sufficient reason to rank it as the top priority for public health interventions. Economic considerations alone cannot be the primary criteria that lead to the choice of the primary prevention strategy vs. the high risk strategy.

## Conclusion

High prevalence of major risks factors was found in a rapidly developing country. Cost for treatment needed to reduce risk factors in all high-risk individuals exceeded resources generally available in low or middle income countries. These findings stress the need for affordable, cost-effective treatment strategies and the critical importance of public health strategies aimed at reducing risk factors in the entire population.

## Competing interests

The author(s) declare that they have no competing interests.

## Authors' contributions

PB was the main investigator of the Seychelles Heart Study and conceptualized and conducted data analysis presented here and lead the writing of this report. CS, AG, WR and FP participated in all aspects of the conceptualization and preparation of the article. All authors read and approved the final manuscript.

## Pre-publication history

The pre-publication history for this paper can be accessed here:



## Figures and Tables

**Table 1:** Prevalence (and 95% confidence intervals) of risk factors in the adult population of Seychelles

	Men				Women				Population aged 25–64*		
			
	25–34	35–44	45–54	55–64	25–34	35–44	45–54	55–64	Men	Women	Total
Smoking	30.2	32.1	31.2	29.3	4.0	4.0	3.9	3.3	30.8	3.9	17.4
	22.7–38.8	24.7–40.5	24.4–38.9	22.6–37.1	1.8–8.7	1.9–8.1	1.9–7.9	1.5–7.2	27.0–34.9	2.6–5.7	15.3–19.7
											
Overweight (BMI ≥25 kg/m^2^)	41.3	55.2	63.3	50.7	55.0	67.6	76.2	83.4	52.0	68.3	60.1
	33.0–50.1	46.7–63.5	55.5–70.5	42.7–58.6	46.9–62.9	60.3–74.1	69.5–81.9	77.3–88.2	47.7–56.2	64.5–71.8	57.2–62.9
Obesity (BMI ≥30 kg/m^2^)	9.5	14.9	22.8	14.7	22.8	37.5	39.8	48.1	15.0	35.2	25.1
	5.5–16.1	9.8–22.1	16.9–30.0	9.8–21.3	16.8–30.3	30.6–44.9	32.9–47.1	40.9–55.4	12.3–18.3	31.6–38.8	22.8–27.5
											
HBP grade ≥1 (≥140/90 mmHg)	20.6	33.6	49.4	64.7	6.0	19.9	39.2	48.6	38.4	24.8	31.6
	14.4-28-6	26.1–42.0	41.6–57.2	56.7-71-9	3.2–11.2	14.6–26,5	32.3–46.5	41.4–55.9	34.6–42.3	22.0–27.9	29.1–34.2
HBP grade ≥2 (≥160/100 mmHg)	5.6	7.5	22.2	24.7	1.3	5.1	16.0	18.8	13.2	8.8	11.0
	2.7–11.2	4.1–13.4	16.3–29.3	18.4–32.2	0.3–5.2	2.7–8.6	11.3–22.1	13.7–25.2	10.8–16.1	7.0–10.8	9.4–12.8
Current treatment for HBP	5.6	14.9	32.9	46.0	6.0	18.2	37.6	58.6	21.4	25.6	23.5
	2.7–11.2	9.8–22.1	26.5–40.7	28.2–54.1	3.2–11.2	13.1–24.6	30.8–44.9	51.2–65.5	18.5–24.6	22.8–28.6	21.4–25.7
HBP grade ≥1 or treatment	23.0	38.8	56.3	72.7	10.1	30.1	51.9	70.2	43.6	35.5	39.6
	15.5–31.2	30.9–47.4	48.5–63.9	65.0–79.2	6.1–16.1	23.8–37.3	44.6–59.2	63.1–76.4	40.0–47.6	32.4–38.8	36.9–42.3
											
TC borderline or high (≥5.2 mmol/l)	41.3	55.2	57.6	53.3	36.2	48.3	60.2	69.6	51.1	50.8	51.0
	33.0–50.0	46.7–63.5	49.7–65.1	45.3–61.2	28.9–44.3	41.0–55.7	52.9–67.1	62.5–75.9	46.8–55.3	47.0–54.6	48.1–53.8
TC high (≥6.2 mmol/)l	17.5	31.3	29.8	26.7	16.1	14.8	32.0	53.6	25.8	25.7	24.2
	11.7–25.2	24.0–39.7	23.1–37.4	20.2–34.4	11.0–23.0	10.2–20.8	25.6–39.2	46.3–60.7	22.3–29.7	22.7–28.9	23.4–28.3
HDL-C low (<1.0 mmol/l)	19.8	31.3	28.5	20.0	14.8	12.5	20.4	21	25.2	16.5	20.8
	13.7–27.8	24.0–39.7	22.0–36.1	14.3–27.2	10.0–21.5	8.4–18.3	15.2–27.0	15.7–27.6	21.6–29.1	13.8–19.5	18.6-23-3
											
Diabetes (FBG≥7.0 mmol/l)	0.8	9.7	12.7	22.0	2.0	4.6	11.6	26.5	9.6	9.0	9.3
	0.1–5.5	5.7–16.0	8.3–18.9	16.1–29.3	0.6–6.1	2.3–8.9	7.7–17.2	20.6–33.4	7.6–12.1	7.3–11.2	7.9–10.9
											
Metabolic syndrome	4.9	19.4	29.1	28.5	9.4	21.6	36.1	48.0	18.7	25.5	22.1
	2.2–10.5	13.3–27.0	22.5–36.7	21.7–36.4	5.6–15.3	16.1–28.3	29.4–43.4	40.8–55.4	15.7–21.9	22.5–28.7	20.0–24.3

HBP: high blood pressure; TC: total cholesterol; HDL-C: high density lipoprotein cholesterol; FBG: fasting blood glucose.

* Overall estimates are adjusted to the distribution of the new World Health Organization standard population (19)

**Table 2:** Medications considered in estimation of costs for treatment for high blood pressure, hypercholesterolemia, and diabetes

		Cost per day (SCR)	
		
	Dose per day	2004	2005
*Daily dose and cost of medications*			
Metformin	1.5 g	4.83	0.48
Glibenclamide	10 mg	1.22	0.18
Insulin (2004:standard; 2005: pen)	20 units	3.40	7.72
Rosuvastatin (2004: Atorvastatin)	10 mg	7.81	7.31
Bendrofluazide (D)	2.5 mg	0.04	0.05
Atenolol (BB)	50 mg	0.67	0.21
Amlodipin (CCB)	5 mg	2.74	0.51
Lisinopril (ACEI)	20 mg	4.00	1.89
Valsartan (ARB)	80 mg	8.50	5.82
*Combinations for treatment of hypertension (mono-, bi-, tri- or quadri-therapy)*			
D in 80% of cases or any of (BB, CCB or ACEI/ARB) in 20% of cases	Mono	0.52	0.21
D + any of (BB or CCB or ACEI/ARB)	Bi	2.51	0.92
D + BB + any of (CCB +ACEI/ARB)	Tri	4.08	1.46
D + BB +CCB + (ACEI/ARB)	Quadri	7.45	2.66
*Average cost of Lisinopril in 90% cases or Valsartan in 10% of cases (ACEI/ARB)*		4.45	2.28
Average cost of either BB or CCB or ACEI/ARB		2.47	0.87
Average cost of either CCB or ACEI/ARB		3.37	1.20

US$1 ~ SCR5

**Table 3:** Distribution of cardiovascular risk categories and estimated costs associated with treatment of hypertension, dyslipidemia and diabetes mellitus

					Cost					
					
					2004			2005		
						
Condition/criterion	Associated conditions	Risk*	Medication	Prevalence in population aged 25–64 (%)	Cost per day per treated patient (SCR)	Annual cost for all cases aged ≥25 yrs (SCR million)	Per capita cost for total population per year (US$)	Cost per day per treated patient (SCR)	Annual cost for all cases aged ≥25 yrs (SCR million)	Per capita cost for total population per year (US$)
**Diabetes (DM)**										
FBG ≥10 mmol/l		High	Metformin 1.5 g/d & Glibenclamide 10 mg/d	4.9	6.05	4.95	12.4	0.66	0.54	1.3
			Addition of insulin 20 U (in 10% of cases)		3.40	0.28	0.7	7.72	0.63	1.6
FBG 7–9 mmol/l		High	Metformin 1.5 g/d	3.6	4.83	2.92	7.3	0.48	0.29	0.7
FBG <7 mmol/l	Treatment for DM	High	Metformin 1 g/d	0.9	3.19	0.45	1.1	0.32	0.05	0.1
*Total diabetes*				*9.3*		*8.60*	*21.5*		*1.51*	*3.8*
**Hypertension**										
≥180/110 mmHg	-	High	2 drugs (20%), 3 drugs (70%), 4 drugs (10%)	3.1	4.10	2.11	5.3	1.47	0.76	1.9
160–179/100–109	≥3 MRF or DM	High	2 drugs (60%), 3 drugs (30%), 4 drugs (10%)	2.3	3.47	1.33	3.3	1.26	0.48	1.2
160–179/100–109	0–2 MRF, not DM	Medium	1 drug (10%), 2 drugs (60%), 3 drugs (30%)	5.6	2.78	2.63	6.6	1.01	0.96	2.4
140–159/90–99	≥3 MRF or DM	High	1 drug (20%), 2 drugs (70%), 3 drugs (10%)	5.6	2.27	2.14	5.4	0.83	0.79	2.0
140–159/90–99	1–2 MRF, not DM	Medium	1 drug (60%), 2 drugs (40%)	12.1	1.32	2.67	6.7	0.50	1.01	2.5
140–159/90–99	0 MRF	Low	-	4.3	0			0		
<140/90	Treatment and ≥1 MRF	≥Medium	1 drug (70%), 2 drugs (30%)	6.9	1.12	1.28	3.2	0.43	0.49	1.2
*Total hypertension*				*39.9*		*12.16*	*30.4*		*4.48*	*11.2*
**High cholesterol**										
≥8 mmol/l		High	Rosuvastatin 10 mg/d (80%), 20 mg (20%)	3.1	9.37	4.80	12.0	8.77	4.49	11.2
7–7.9 mmol/l	≥2 MRF or DM	High	Rosuvastatin 10 mg/d	3.5	7.81	4.56	11.4	7.31	4.27	10.7
6–6.9 mmol/l	≥3 MRF or DM	High	Rosuvastatin 10 mg/d	2.8	7.81	3.71	9.3	7.31	3.47	8.7
*Total dyslipidemia*				*9.4*		*13.07*	*32.7*		*12.24*	*30.6*
**Any condition**				*42.7*		*33.8*	*84.6*		*18.22*	*45.6*

*CVD risk assessed based on following risk factors: age ≥55 for men; smoking; total cholesterol ≥6.2 mmol/l (240 mg/dl); HDL-cholesterol <1.0 mmol/l (40 mg/dl), BP ≥140/90 mmHg or treatment, obesity (body mass index ≥30 kg/m^2^); SCR: Seychelles rupees.

**Table 4:** Estimated costs of medical care for hypertension, diabetes and dyslipidemia

	Unit cost (SCR)	Number done each year (n)	Cost per year (SCR)	Prevalence in population aged 25–64 (%)	Cost in population per year (SCR million)	Cost per capita and per year (US$)
Medical consultations	42	4	168	42.7	3.3	8.24
Blood tests for all persons with hypertension, diabetes or high cholesterol						
Glucose	30	1	30	42.7	0.6	1.47
Creatinine	30	1	30	42.7	0.6	1.47
Sodium	30	1	30	42.7	0.6	1.47
Potassium	30	1	30	42.7	0.6	1.47
Total cholesterol	30	1	30	42.7	0.6	1.47
HDL-cholesterol	50	1	50	42.7	1.0	2.45
Triglycerides	30	1	30	42.7	0.6	1.47
*Subtotal*				*42.7*	*6.2*	*11.3*
Additional blood tests for all persons with diabetes						
Glucose	30	3	90	9.3	0.4	0.96
HbA1c	100	2	200	*9.3*	0.9	2.14
*Subtotal*					*1.2*	*3.1*
*All blood tests*					*7.5*	*14.4*
*All blood tests and consultations*					*10.8*	*22.6*

SCR: Seychelles rupees
